# Postbiotic Activities of *Bifidobacterium adolescentis*: Impacts on Viability, Structural Integrity, and Cell Death Markers in Human Intestinal C2BBe1 Cells

**DOI:** 10.3390/pathogens13010017

**Published:** 2023-12-23

**Authors:** María Hernández, Martin Sieger, Alfonso Barreto, Carlos A. Guerrero, Juan Ulloa

**Affiliations:** 1 Laboratorio de Virología, Grupo de Enfermedades Infecciosas, Facultad de Ciencias, Pontificia Universidad Javeriana, Bogotá 110231, Colombia; he-maria@javeriana.edu.co (M.H.); m-sieger@javeriana.edu.co (M.S.); 2 Grupo de Inmunobiología y Biología Celular, Departamento de Microbiología, Facultad de Ciencias, Pontificia Universidad Javeriana, Bogotá 110231, Colombia; alfonso.barreto@javeriana.edu.co; 3 Laboratorio de Biología Molecular de Virus, Facultad de Medicina, Universidad Nacional de Colombia, Bogotá 111311, Colombia

**Keywords:** Bifidobacteria, *Bifidobacterium adolescentis*, anti-rotavirus, in vitro toxicity testing

## Abstract

Acute diarrheal disease (ADD) caused by rotavirus (RV) contributes significantly to morbidity and mortality in children under five years of age. Currently, there are no specific drugs for the treatment of RV infections. Previously, we reported the anti-rotaviral activity of the protein metabolites derived from *Bifidobacterium adolescentis*. In this study, our aim was to assess the impact of *B. adolescentis*-secreted proteins (BaSP), with anti-rotaviral activity on the human intestinal C2BBe1 cell line. We initiated the production of BaSP and subsequently confirmed its anti-rotaviral activity by counting the infectious foci using immunocytochemistry. We then exposed the C2BBe1 cells to various concentrations of BaSP (≤250 µg/mL) for 72 h. Cell viability was assessed using the MTT assay, cell monolayer integrity was monitored through transepithelial electrical resistance (TEER), and cytoskeleton architecture and tight junctions (TJs) were examined using confocal microscopy with F-actin and occludin staining. Finally, we utilized a commercial kit to detect markers of apoptosis and necrosis after 24 h of treatment. The results demonstrated that BaSP does not have adverse effects on C2BBe1 cells. These findings confirm that BaSP inhibits rotavirus infectivity and has the potential to strengthen intestinal defense against viral and bacterial infections via the paracellular route.

## 1. Introduction

Acute diarrheal disease (ADD) is a public health problem worldwide and is the second leading cause of death in children under five years of age [1]. The primary infectious agent of ADD is rotavirus (RV); however, other microorganisms such as *Escherichia coli*, *Shigella spp*, and *Cryptosporidium* also play an important role [1,2]. Each year, ADD reaches approximately 1.7 billion cases and causes the death of about 525,000 children under five years of age [1]. ADD caused by RV is characterized by symptoms such as mild to severe diarrhea, vomiting, and varying degrees of dehydration. Fever, when present, is typically mild. Vomiting often precedes diarrhea and is of shorter duration, while diarrhea can persist for several days. Dehydration, often severe, is a common complication and can lead to significant electrolyte imbalances [3]. Although live-attenuated RV vaccines are available, they are not accessible to 40% of the worldwide child population. In addition, new RVs have emerged through genetic reassortment, which can generate antigenic variability [4,5].

The recommended treatment for ADD is administering oral rehydration solutions to replace lost fluids [1,6]; however, this is not a specific therapy for RV infection. In recent years, probiotics have emerged as a therapeutic alternative for combatting RV infection and have shown positive effects in managing RV diarrhea [7,8]. The use of some species, such as *L. rhamnosus*, *S. boulardii*, *L. acidophilus*, *B. longum*, *B. lactis*, and *B. adolescentis*, has resulted in a significant reduction in the length and severity of rotavirus diarrhea, as well as in the incidence of RV infection, and has been shown to modulate the immune response of affected children [9,10,11,12,13,14]. Some in vitro studies have shown that the activity of *B. adolescentis* against several DNA and RNA viruses is mediated by different mechanisms, such as the regulation of gene expression, possible hindrance of viral adsorption, and production of metabolites (postbiotics) with direct antiviral effects [15,16,17,18,19]. Our research group has compared the antirotaviral effects of several probiotics belonging to the Bifidobacterium and Lactobacillus genera [20,21]. The results obtained indicate that the effective antirotaviral activity of *B. adolescentis* is attributed to direct interactions between the bacterial secretome proteins and the infectious rotavirus particles. 

Thus, *B. adolescentis*-secreted proteins (BaSP) represent a possible alternative for preventing and treating rotavirus infection. However, studies on the potential use of these proteins in humans must include toxicity testing to determine their potential adverse effects on the intestinal epithelial cells (IECs) [22]. IECs constitute the intestinal epithelial barrier, the first and largest protective barrier against exogenous molecules [23]. This barrier plays an essential role in the integrity of the host intestinal mucosa [19] by maintaining intestinal homeostasis and an effective immune response [20]. Any effect of exogenous compounds that compromise the integrity of IECs can lead to different disorders, such as inflammatory bowel disease, necrotizing enterocolitis, and irritable bowel syndrome [24]. 

Determining the potentially toxic effects of substances on human IECs requires using tumor cell lines, of which the most commonly used is HT-29 as well as Caco-2 and their clones [25]. These cell lines are very diverse in terms of type and differentiation status, proliferation potential, and metabolic properties; some exhibit characteristics of differentiated enterocytes or mucosal cells [25,26]. C2BBe1 cells, which are subclones derived from the human colon adenocarcinoma cell line Caco2, are commonly employed due to their resemblance to human IECs [27]. C2BBe1 cells exhibit characteristics of differentiated enterocytes, have an apical brush border like human intestinal cells, form polarized monolayers in culture [27], and can synthesize tight junction proteins [28]. Strong tight junctions (TJs) prevent the paracellular diffusion of microorganisms, antigens, and xenobiotics across the epithelial barrier [29,30]. The use of cells that mimic human physiology also allows the study of substances that induce cellular stress markers, such as ROS, DNA fragmentation, and changes in the cytoskeleton structure, among others [31,32]. Considering that BaSP represents an alternative for the prevention and treatment of diarrhea caused by rotavirus, it is important to predict the potential effects they may have in an in vitro intestinal model, such as the C2BBe1 cell line.

## 2. Materials and Methods

### 2.1. Production of the BaSP Pool 

*Bifidobacterium adolescentis* was purchased from the German Collection of Microorganisms and Cell Cultures GmbH (DSMZ GmbH). The lyophilized bacteria were resuspended in 1 mL of MRS broth (Man–Rogosa–Sharpe, Oxoid, Basingstoke, UK) and incubated at 37 °C for 48 h under anaerobic conditions (activation phase). After this period, 0.1 mL of *B. adolescentis* was transferred in triplicate to a solid MRS medium and incubated under the same conditions for 48 h to count the colony-forming units per milliliter (CFU/mL). Finally, the identity and purity of the culture were verified using the commercial BBL™ Crystal™ ANR ID System (Becton Dickinson, Sparks, MD, USA) following the manufacturer’s instructions and using Gram staining.

To characterize the growth of *B. adolescentis* and determine the logarithmic phase in a 500 mL batch, the bacteria were initially cultured on a solid MRS medium as described. Subsequently, several colonies were resuspended in 10 mL of phosphate-buffered saline (1× PBS) to achieve a concentration equivalent to the McFarland standard tube #1. Following this, they were inoculated into 500 mL of MRS broth and incubated for 48 h under the same conditions. Optical density measurements were taken at a wavelength of 540 nm every 6 h for a total duration of 48 h. In total, two batches of the bacteria culture were produced, and the averages of the results were graphed and analyzed using GraphPad Prism 6.0b software for Windows 10 (GraphPad, San Diego, CA, USA). 

Once the logarithmic phase was determined, the bacteria were cultured again for an incubation period of 30 h. After this time, the culture was centrifuged at 730× *g* for 30 min at 4 °C. The supernatant containing the primary metabolites produced was recovered and filtered using a 0.22 μm pore size filter (TPP^®^) to remove residual bacteria. Next, the metabolites were separated and concentrated by molecular size through ultrafiltration using a Millipore Masterflex peristaltic pump (Millipore, Bedford, MA, USA) with a Pellicon^®^ XL 50 ultrafiltration cassette (Merck KGaA, Darmstadt, Germany) that had a 1 kDa cutoff. The retained metabolites were then mixed with polyethylene glycol (PEG 8000) at a concentration of 10% *w/v* to precipitate the proteins [33,34]. This mixture was left overnight (16 h) under constant stirring at 4 °C. After this period, the mixture was centrifuged at 16,000× *g* for 30 min at 4 °C. The supernatant was carefully removed, and the pellet was resuspended in 25 mL of 1× PBS. The concentrated metabolite pool was stored at 4 °C until use.

The final protein concentration was quantified using the Pierce™ BCA Protein Assay Kit (Thermo Fisher Scientific, Waltham, MA, USA), following the manufacturer’s instructions. In addition, protein presence was confirmed using polyacrylamide gel electrophoresis (PAGE) under native conditions rather than denaturing conditions, as problems stemming from the interaction between PEG and sodium dodecyl sulfate (SDS) in SDS-PAGE [35] have been reported. Finally, the proteins were detected with silver staining.

### 2.2. Verification of the Anti-Rotaviral Activity of the BaSP

Before assessing the anti-rotaviral activity of the BaSP pool, the maximum non-toxic concentration (MNTC: maximum concentration determining cell viability ≥ 95%) was defined using the MA-104 cells. For this, the MA-104 cells were cultured in 96-well plates with Advanced DMEM (Dulbecco’s Modified Eagle Medium, Invitrogen™; Grand Island, NY, USA) supplemented with 5% fetal bovine serum (FBS), 2 mM L-glutamine, and antibiotics/antimycotic for 48 h at 37 °C and 5% CO_2_ atmosphere. Cells were then exposed independently to different concentrations of the BaSP pool from Batch 1, which had the highest concentration, ranging from 0.03 µg/mL to 250 µg/mL (2×-serial dilutions in culture medium without FBS) for 24 h. Cell viability was determined using the MTT (3-(4,5-dimethylthiazol-2-yl)-2,5-diphenyltetrazolium; (Sigma Aldrich Co., Ltd.; St, Louis, MO, USA) colorimetric assay (30 μL of 1 mg/mL MTT per well) according to the manufacturer’s instructions. The MA-104 cells that were unexposed to the BaSP pool (cell control: CC) and were instead exposed to 1 mM H_2_O_2_ (cytotoxicity control) were cultured in parallel. Additionally, an abiotic control was included that consisted of the treatment of MA-104 cells with *B. adolescentis*-free MRS medium proteins (MRS-P), precipitated with PEG 8000 using the same evaluated concentrations of the BaSP. The abs at 540 nm were read using a MultisKan™ FC Microplate Photometer (Thermo Scientific™). The entire experiment was carried out in triplicate and repeated three times. The percentage of cell viability was calculated using the following equation:

% of cell viability = [100 × (mean Abs of the sample)/(mean Abs of cell control)](1)

Once the MNTC and the non-cytotoxic concentrations of the BaSP and the MRS-P pools were determined, their anti-rotaviral activity was evaluated. 

For this purpose, the rotavirus (Rhesus Rotavirus: RRV) particles were initially activated with 10 µg/mL trypsin for 1 h at 37 °C, and then the trypsin was neutralized with trypsin inhibitor (Gibco^®^ Life Technologies, Grand Island, NY, USA) for 5 min at room temperature (RT). The infectious RVs were then mixed at a final multiplicity of infection (MOI) of 0.1 with the non-cytotoxic concentrations of the BaSP pool and incubated for 1 h at 37 °C. These mixtures were added in triplicate to the MA-104 cells grown in 96-well plates as described above and incubated for 1 h at 37 °C in a 5% CO_2_ atmosphere. In addition, the cells infected with RRV (positive infectivity control), the cells not infected with RRV (mock control), and the cells exposed to the infectious RVs previously mixed and incubated for 1 h at 37 °C with the MNTC of the MRS-P were cultured in parallel. Subsequently, inocula were removed, two washes with 1× PBS were carried out, and fresh medium without FBS was added. Cells were incubated for an additional 9 h and immunocytochemically stained for RV TLPs, as described by Tellez in 2015 [36]. Next, focus-forming units (FFU) (positive for RV TLPs) were counted using an Olympus CKX41 microscope (Olympus Instruments, Tokyo, Japan) with a 20× objective. The percentage of viral infectivity was calculated in relation to the positive infectivity control. Micrographs were captured with a 10× objective using a Moticam 10+ camera (Motic^®^; Kowloon, Hong Kong) coupled to an Olympus CKX41 microscope (Olympus Instruments, Tokyo, Japan). The entire experiment was repeated three times.

### 2.3. Evaluation of the Effects of BaSP on Viability, Structure, and Expression of Death Markers of the Human Intestinal C2BBe1 Cell Line

#### 2.3.1. Evaluation of Cell Viability

C2BBe1 cells (ATCC 2102™, Manassas, VA, USA) were grown in 96-well plates in Advanced DMEM supplemented with 5% FBS, 2 mM L-glutamine, and antibiotics/antimycotic for 72 h up to 90% of confluence at 37 °C in a 5% CO_2_ atmosphere. These cell cultures were then exposed independently to different concentrations of the BaSP pool, ranging from 0.03 µg/mL to 250 µg/mL (2×-serial dilutions in advanced DMEM without FBS) for 72 h. Cell viability was determined by the MTT assay (50 μL of 1 mg/mL MTT per well) according to the manufacturer’s instructions. Cells unexposed to the BaSP pool in medium without FBS (cell control: CC) and cells exposed to 50 mM H_2_O_2_ (positive cytotoxicity control) were cultured in parallel. The entire experiment was carried out in triplicate and repeated three times. The percentage of cell viability was calculated as described above.

#### 2.3.2. Transepithelial Electrical Resistance (TEER) 

C2BBe1 cells (1 × 10^4^ per well) were seeded and cultured, as described above, in Transwell^®^ 12-well permeable supports (Costar^®^, Washington, DC, USA; Cat # 3401). The culture medium was changed every 4 days. TEER was measured with a Millicell^®^-ERS (Millipore, Bedford, MA, USA) until it reached a value of 300 Ω/cm^2^ (14–21 days) to confirm the differentiation, polarization, and integrity of cell monolayers [37]. Subsequently, cells were washed with Advanced DMEM without FBS and independently exposed to different concentrations of the BaSP pool ranging from 0.98 µg/mL to 250 µg/mL (4×-serial dilutions in Advanced DMEM without FBS) for 72 h. TEER values were measured every 24 h. The entire experiment was carried out in triplicate and repeated three times.

#### 2.3.3. Architecture of the Cytoskeleton and Tight Junctions

The confluent C2BBe1 cell monolayers from the above assay were stained with Alexa Fluor^®®^ 594-conjugated anti-occludin mAb (OC-3F10; Thermo Fisher Cat # 331594) and Alexa Fluor^®®^ 488-conjugated phalloidin (Molecular Probes™ Cat # 12379, Eugene, OR, USA) for F-actin detection, according to the respective manufacturer’s instructions. Briefly, the cell monolayers were washed twice with 1× PBS and fixed with 2% paraformaldehyde in 1× PBS for 15 m at room temperature (RT). Next, cells were incubated with 1% bovine serum albumin (BSA) in 1× PBS (30 m at RT), permeabilized with 0.03% Triton ×-100 in 1× PBS (5 m), stained with Alexa Fluor^®®^ 594-conjugated anti-occludin mAb (1 µg/mL in 1% BSA in 1× PBS) (3 h at RT) and with Alexa Fluor^®®^ 488-conjugated phalloidin prepared according to the manufacturer’s instructions, and then diluted in 1% BSA in 1× PBS (30 m at RT). Each step was followed by two washes with 1× PBS, except after incubation with phalloidin, which was followed by three washes. ProLong™ Gold Antifade Mountant (Thermo Fisher, Cat # P36930) was added. Confocal fluorescent images (saved as 640 × 640 resolution bitmaps for later analyses) were captured using an Olympus FV 1000 confocal microscope (Olympus, Tokyo, Japan) equipped with lasers providing excitations at 488 nm (for F-actin) and 543 nm (for occludin). The images were captured using a UPLSAPO, 60 × NA 1.35. For the Z-series, 0.15-µm slice spacing was used.

#### 2.3.4. Detection of Cell Death Markers: Apoptosis and Necrosis

Cell death was assessed using the commercial ApoDETECT™ Annexin V-FITC Kit (Invitrogen™; Cat # 331200) following the manufacturer’s instructions and quantifying fluorescence by fluorometry. Thus, the C2BBe1 cells (1 × 10^4^ per well) were cultured in 96-well black bottom plates (Costar^®^, Cat # 3603), as described above, for 14 days until polarization. On day 14, the cells were exposed to different concentrations of the BaSP pool ranging from 0.98 µg/mL to 250 µg/mL (4×-serial dilutions in advanced DMEM without FBS) for 24 h. After three washes with 1× PBS and following the manufacturer’s instructions, the cells were incubated with the kit reagents, i.e., FITC-conjugated Annexin V and propidium iodide (20 μg/mL), in darkness. The fluorescence was quantified as relative fluorescence units (RFU) using a FLUOStar^®^ Omega Plate Reader (BMG LABTECH, Ortenberg, Germany) equipped with 488 nm and 617 nm filters. The entire experiment was carried out in triplicate and repeated three times.

#### 2.3.5. Statistics

Data were analyzed using GraphPad Prism 6.0b software for Windows 10 (GraphPad, San Diego, CA, USA). For significance testing, the distributions were determined using Shapiro-Wilk normality tests. Analyzes of all assays were performed using the nonparametric Mann-Whitney U test to determine the differences between different concentrations of the BaSP or MRS-P and the cellular control or infectivity control.

## 3. Results

### 3.1. BaSP Pool Production

**Figure 1:**
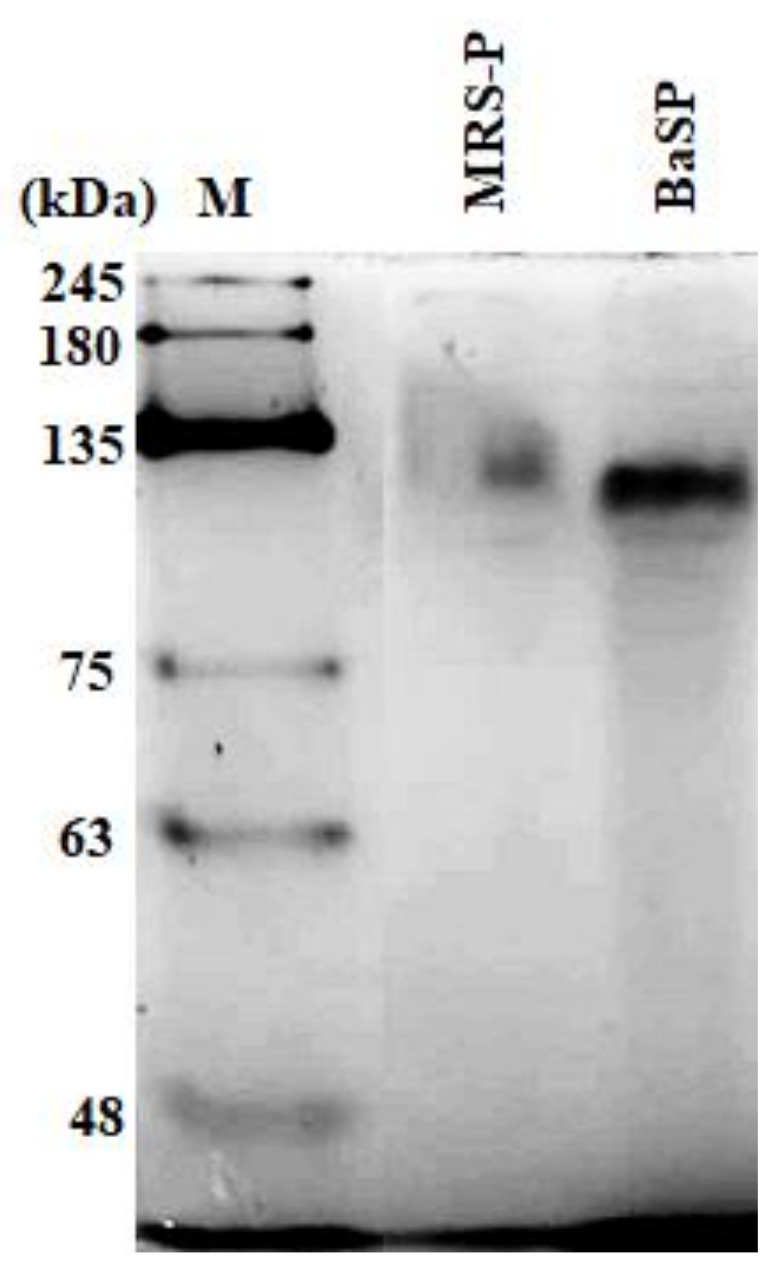
Polyacrylamide gel electrophoresis under native conditions (7.5% PAGE) to confirm the presence of *B. adolescentis*-secreted proteins (BaSP). From right to left, molecular weight protein marker (M), *B. adolescentis*-free MRS medium proteins (MRS-P), and BaSP from Batch 1. 100 ng of each protein pool was loaded in each lane. Proteins were stained with silver nitrate.

The concentration of BaSP obtained from the ultrafiltration and precipitation processes of the two batches of *B. adolescentis* culture was quantified, resulting in 3898 µg/mL for Batch 1 and 2048 µg/mL for Batch 2. The concentration factor was 20, resulting in a final volume of 25 mL. In addition, the presence of BaSP was confirmed with 7.5% polyacrylamide gel electrophoresis (PAGE) under native conditions. Figure 1 shows the detection of BaSP from Batch 1 and MRS-P (abiotic control), with which the subsequent experiments were carried out.

### 3.2. Anti-Rotaviral Activity of BaSP

**Figure 2:**
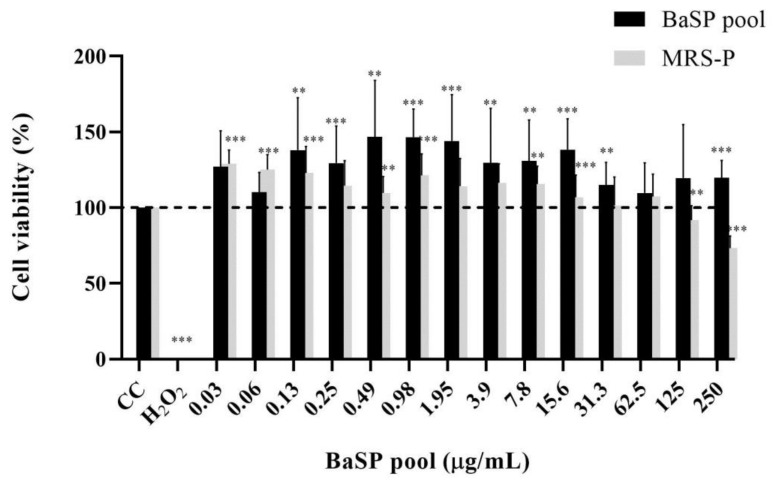
Effects of *B. adolescentis*-secreted proteins (BaSP) and *B. adolescentis*-free MRS medium proteins (MRS-P) on the viability of the MA-104 cell line. The MA-104 cells were treated with different concentrations of the BaSP pool and MRS-P for 24 h, and the cell viability was assessed using the MTT colorimetric (Abs_540nm_) assay. Each bar represents the mean percentage of cell viability for each experimental condition. Error bars represent the standard deviation. Mann-Whitney U test for comparisons with the CC; *** *p* ≤ 0.004, ** and *p* ≤ 0.0088. CC: cell control. H_2_O_2_: positive cytotoxicity control (*n* = 3 independent experiments).

Before assessing the anti-rotaviral activity of the BaSP pool (Batch 1), its MNTC was determined using MA-104 cells. None of the concentrations tested (0.03 μg/mL to 250 μg/mL) decreased the percentage of cell viability; therefore, 250 µg/mL was considered the MNTC of the BaSP pool (Figure 2). Furthermore, the percentage of cell viability observed in most concentrations of the BaSP pool was significantly higher than the 100% viability in the cell control. In the case of MRS-P, concentrations of 125 µg/mL and 250 µg/mL significantly reduced the viability of MA-104 cells. The MNTC was 62.5 µg/mL.

**Figure 3:**
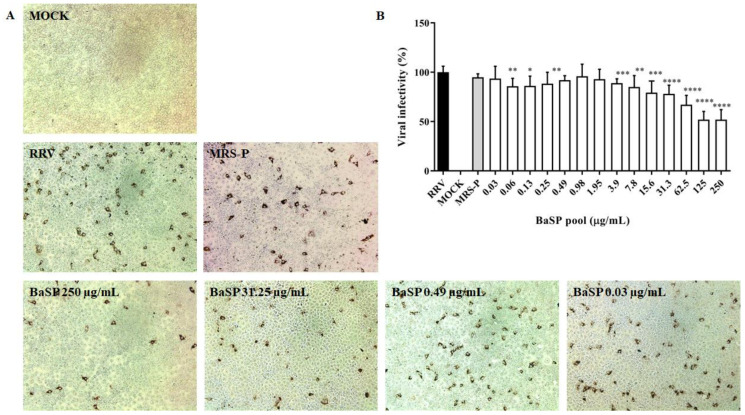
Anti-rotaviral (virucidal) effect of *B. adolescentis*-secreted proteins (BaSP). The confluent monolayers of the MA-104 cells were exposed to rotavirus (Rhesus Rotavirus: RRV) suspensions (MOI = 0.1) mixed with different concentrations of the BaSP pool for 1 h, washed, and additionally incubated for 9 h. Next, cytoplasmic RV structural proteins (outer capsid) were immunocytochemically detected, FFUs were counted, and the percentage of viral infectivity was calculated based on the positive infectivity control. (**A**) Representative micrographs of FFU (positive for RV outer capsid structural proteins) were captured with a Moticam 10+ camera using a 10X objective. (**B**) Bar chart representing the percentage of viral infectivity. Bars represent mean percentages of infectivity, and the error bars represent standard deviations. * *p* ≤ 0.0106, ** *p* ≤ 0.0072, *** *p* ≤ 0.0003, and **** *p* ≤ 0.0001; Mann-Whitney U Test for comparisons with the positive infectivity control. RRV: RRV-infected cells, positive infectivity control. MOCK: RRV-uninfected cells. MRS-P: *B. adolescentis*-free MRS medium proteins (MNTC = 62.5 µg/mL) were cultured in parallel (*n* = 3 independent experiments).

As none of the BaSP pool concentrations evaluated decreased the cell viability of the MA-104 cells, their anti-rotaviral activity was tested. Most of the BaSP pool concentrations significantly reduced the RRV infectivity in MA-104 cells as compared to the positive infectivity control. The highest percentages of infectivity reduction were observed with 125 µg/mL (49.58%) and 250 µg/mL (49.55%) of the BaSP pool (Figure 3). The MNTC of MRS-P did not exert anti-rotaviral activity.

### 3.3. Effects of BaSP on Viabilty, Structure, and Expression of Death Markers of the Human Intestinal C2BBe1 Cell Line

**Figure 4:**
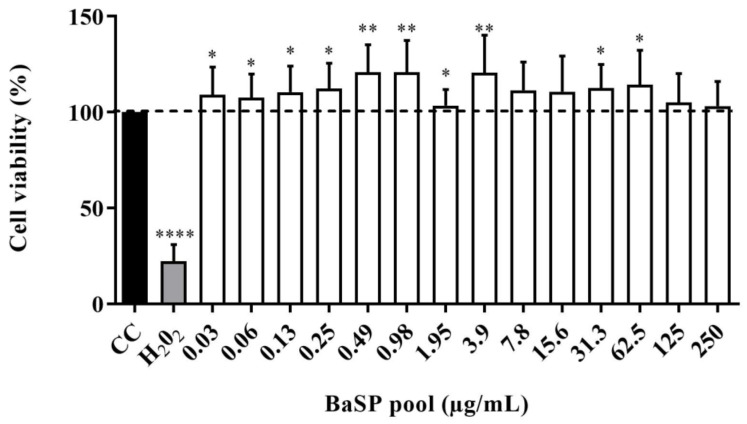
Effect of *B. adolescentis*-secreted proteins (BaSP) on the viability of the C2BBe1 cell line. The C2BBe1 cells were treated with different concentrations of the BaSP pool for 72 h. Next, the cell viability was assessed using the MTT colorimetric (Abs_540nm_) assay. Bars represent the mean percentages of cell viability, and the error bars represent standard deviations. * *p* ≤ 0.0361, ** *p* ≤ 0.0019, ****, *p* ≤ 0.0001; Mann-Whitney U test for comparisons with the CC. CC: cell control. H_2_O_2_: positive cytotoxicity control (*n* = 3 independent experiments).

The viability of C2BBe1 cells exposed to different concentrations of the BaSP pool ranging from 0.03 µg/mL to 250 µg/mL was evaluated using the MTT colorimetric assay. None of the concentrations tested decreased significantly (*p* < 0.05) the percentage of cell viability compared to the cell control (untreated cells). Moreover, in cultures exposed to most BaSP pool concentrations, cell viability percentages were significantly higher than 100% in the cell control (Figure 4).

### 3.4. Effect of BaSP on Monolayer Integrity, F-actin Distribution, and Occludin Expression in the C2BBe1 Cell Line

**Figure 5:**
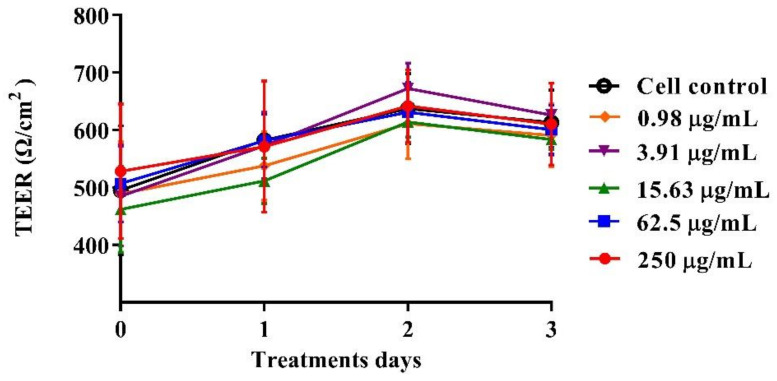
Effect of *B. adolescentis*-secreted proteins (BaSP) on the integrity of the C2BBe1 cell monolayers. The polarized C2BBe1 cells were treated with different concentrations of the BaSP pool, ranging from 0.98 µg/mL to 250 µg/mL for 72 h. The TEER was measured every 24 h. Symbols represent the mean TEER values, and error bars represent standard deviations. CC: cell control. The Mann-Whitney U test was used for comparisons with the cell control (*n* = 3 independent experiments).

**Figure 6:**
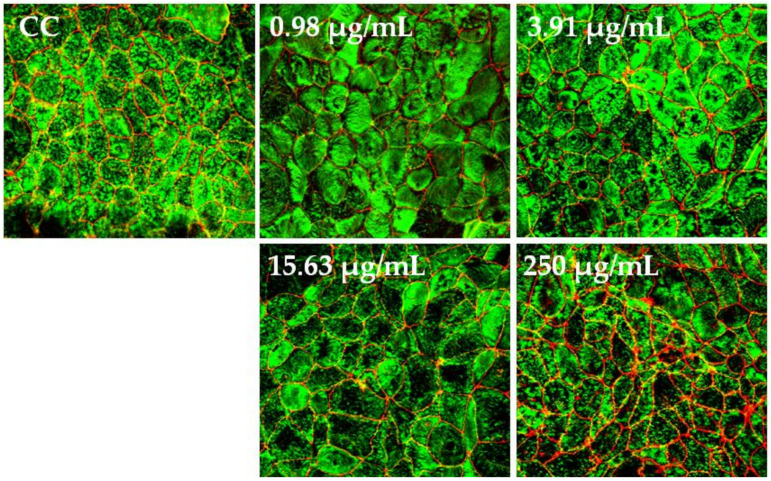
Effect of *B. adolescentis*-secreted proteins (BaSP) on the distribution of F-actin and occludin in C2BBe1 cell monolayers. Confocal images of C2BBe1 cell monolayers exposed to the BaSP pool (0.98 µg/mL to 250 µg/mL) for 72 h and stained with Alexa Fluor^®^ 488-conjugated phalloidin (green) and Alexa Fluor^®^ 594-conjugated anti-occludin mAb (red). Cell control (CC: untreated cells). Magnification, 60×.

The polarized C2BBe1 cell monolayers were exposed to BaSP pool concentrations ranging from 0.98 µg/mL to 250 µg/mL for 72 h. The integrity of the cell monolayers was initially assessed by measuring the TEER and subsequently assessed by evaluation of F-actin distribution and occludin expression. The TEER values of treated and untreated cell monolayers were always greater than 300 Ω/cm^2^, which is indicative of adequate cell monolayer integrity (Figure 5). However, the TEER values tended to be lower (*p* > 0.05) between the second and third days of culture (*p* > 0.05). Finally, none of the treatments carried out with BaSP showed changes related to the loss of integrity of the C2BBe1 cell monolayers, the distribution of F-actin, or the expression of occludin. In particular, cells treated with 250 µg/mL showed greater expression of occludin as compared to both the untreated control and the cells treated with lower concentrations (Figure 6).

### 3.5. Effect of BaSP on the Expression of Cell Death Markers in C2BBe1 Cells: Apoptosis and Necrosis

**Figure 7:**
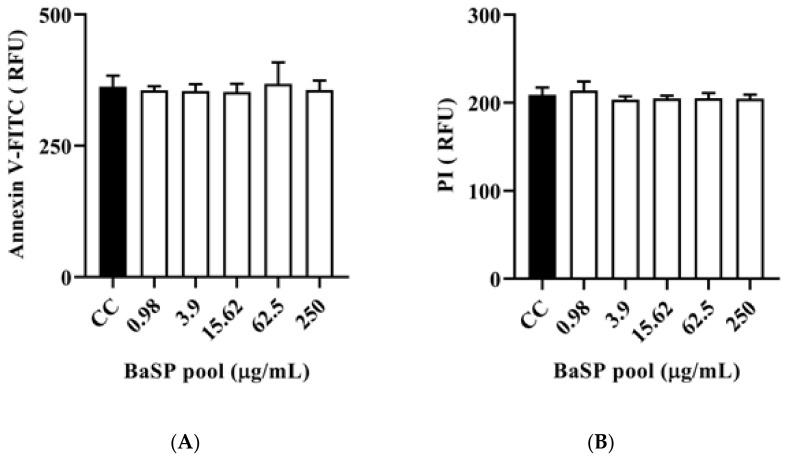
Effect of *B. adolescentis*-secreted proteins (BaSP) on the expression of cell death markers in C2BBe1 cells. The polarized C2BBe1 cell monolayers were exposed to different concentrations of the BaSP pool for 24 h. The ells were then stained with the ApoDETECT™ Annexin V-FITC kit to assess apoptosis and necrosis by fluorometry. The bar graphs show relative fluorescence units (RFU) for (**A**) Annexin V-FITC (488 nm filter) and (**B**) PI (617 nm filter). Bars represent the mean RFU values, and the error bars correspond to standard deviations. Mann-Whitney U test. CC: cell control. (*n* = 3 independent experiments).

The polarized C2BBe1 cell monolayers exposed to BaSP pool concentrations ranging from 0.98 µg/mL to 250 µg/mL for 72 h were stained with the ApoDETECT™ Annexin V-FITC kit to evaluate features of apoptosis (loss of cell membrane asymmetry by binding of Annexin V-FITC to phosphatidylserine in the outer leaflet of the cell membrane) and necrosis (DNA staining after PI entering through a damaged cell membrane). None of the BaSP pool concentrations induced any loss of cell membrane asymmetry or disrupted the cell plasma membrane when compared to the cell control (Figure 7).

## 4. Discussion

In recent decades, the use of probiotics as an alternative for preventing and treating ADD caused by viruses such as RV has been increasing [7,10,38,39]. The bifidobacteria group is among the most studied probiotics for that purpose [8,40]. Some species of the genus *Bifidobacterium* are beneficial in preventing rotavirus infection [41,42], strengthening the intestinal barrier [43], and modulating the immune response [44,45]. Notably, studies on bifidobacteria involve the characterization of the direct effects of specific species and the mechanisms attributed to the bioactive substances they secrete [21,46].

This study aimed to evaluate the effects of anti-rotaviral BaSP on cell viability, cytoskeleton structure (F-actin distribution), TJs (Occludin expression), and the generation of apoptosis and necrosis markers in the human intestinal C2BBe1 cell line. For this purpose, a BaSP pool was obtained from a batch culture of *B. adolescentis*. The cell viability was initially evaluated on MA-104 cells, and it was observed that concentrations ≤ 250 µg/mL did not reduce cell viability (Figure 2). This finding is relevant for the development of antivirals to obtain a good selectivity index (cytotoxic concentration 50 (CC50)/inhibitory concentration 50 (IC50) ratio).

Subsequently, the evaluation of the anti-rotaviral activity of the BaSP pool showed an IC50 of 125 µg/mL (Figure 3). Unfortunately, the protein content of the BaSP pool was insufficient to evaluate higher concentrations in order to determine the CC50; therefore, the SI could not be calculated.

Our research group previously reported that BaSP can reduce RV infection in vitro by directly interacting with infectious virions. Additionally, it decreases intracellular levels of the viral enterotoxin NSP4 and intracellular Ca^+2^ post-infection, both of which are critical for viral maturation and pathogenesis [20,21]. Other preliminary studies from our group suggest that BaSP mainly interacts with the rotavirus structural protein VP5, which is involved in host cell entry. The antiviral activity of *B. adolescentis* has been found in other models of infections with human papillomavirus [15], coxsackie virus [18], hepatitis B virus [16], herpes virus [47], and vesicular stomatitis virus [17]. In addition, other Bifidobacterium species, such as *B. longum*, *B. breve*, and *B. thermophilum,* can also reduce rotavirus infection [46,48,49].

The primary target cells of rotaviruses are mature enterocytes [3]. These epithelial cells constitute the luminal surface of the intestine, which forms the barrier responsible for absorbing and eliminating molecules derived from orally ingested compounds in food, drugs, and xenobiotics [24]. Therefore, molecules such as bacterial metabolites, e.g., BaSP, that are produced in laboratories or industries should be evaluated for their potential effects on the intestines using in vitro tests in the first instance. If a xenobiotic crosses or damages the intestinal barrier, it will cause injury to organs such as the liver, thus being potentially lethal [50].

Based on our previous findings on the anti-rotaviral activity of BaSP, we decided to evaluate their potentially deleterious effects on the C2BBe1 cell line. First, cell viability was assessed in terms of metabolic activity using the MTT test. None of the BaSP pool concentrations that were tested reduced the viability of the C2BBe1 cells (Figure 4). These results correlated with those observed in the MA-104 cells and were in agreement with previous reports showing percentages of cell viability above 95% after exposing cells to *B. adolescentis* [18,41]; however, it should be noted that the biological effects of probiotics depend not only on the species but also on the strain tested [51,52].

Interestingly, the C2BBe1 cultures exposed to most concentrations of the BaSP pool showed a percentage of cell viability higher than 100%. This finding could be related to an increase in cell proliferation. A similar effect has been observed with two extracellular proteins purified from *L. rhamnosus* that activated Akt, inhibited cytokine-induced apoptosis, and promoted epithelial cell growth [53]. Another study reported that stromal cell proliferation was significantly increased in the presence of *B. bifidum*, either in the culture-derived supernatant or the bacterial cell mass itself [54].

Several effects of probiotics may be mediated by the antimicrobial compounds they secrete, a circumstance that can strengthen the epithelial barrier function [55] and rearrange the cytoskeleton and the intercellular junctions [56]. In the present study, the BaSP were shown to neither alter nor adversely affect the integrity of the C2BBe1 cell monolayer or the rearrangement of the cytoskeleton and cellular TJs (Figure 5 and Figure 6). In all experimental conditions, the complete polarization and integrity of the C2BBe1 monolayers were evidenced by TEER values above 300 Ω/cm^2^ (Figure 5). These data are in the range of TEER values previously reported for the CaCo2 cells that vary from 300 Ω/cm^2^ to 2000 Ω/cm^2^ [57].

Furthermore, BaSP did not affect the cytoskeleton architecture of the C2BBe1 cells, as demonstrated by the distribution of F-actin that was similar to that of the untreated cell control (Figure 6). On the other hand, occludin showed augmented fluorescence intensity in the C2BBe1 cell cultures exposed to 250 µg/mL of the BaSP pool (Figure 6). A higher occludin expression may translate into reduced paracellular permeability and, thus, decreased transit of pathogenic bacteria and viruses across the intestinal barrier.

Cell death is an important outcome of cytotoxicity and depends on biological processes defined by biochemical pathways. Cell death can occur through apoptosis or necrosis, each of which is characterized by particular phenomena. For example, apoptosis is characterized by the exposure of phosphatidylserine to the outer leaflet of the plasma membrane (detectable by binding to Annexin V) [58]. In turn, necrosis is characterized by cell membrane damage, which is detectable by staining with supravital dyes such as PI that penetrate the cell and stain nuclear DNA. Figure 7A,B shows no markers of apoptosis or necrosis in C2BBe1 cells exposed to BaSP. Notably, some studies have reported that *B. bifidum* can downregulate apoptosis in vitro and in vivo [59], suggesting a protective mechanism for the host.

In vitro toxicity testing entails exposing cells to a test compound for a duration ranging from 6 to 72 h. This model is primarily focused on assessing acute toxicity and providing data for hazard identification and concentration-effect responses to establish a quantitative starting point. These findings can then be used for biokinetic extrapolation calculations in in vivo tests [60]. In this regard, the data obtained from the tests conducted in this study can serve as a basis for conducting acute toxicity tests in animals. In summary, the results presented in this study demonstrate that anti-rotaviral BaSP do not induce metabolic and structural damage to human intestinal C2BBe1 cells in vitro. These findings also suggest that BaSP may enhance the barrier function of the intestinal epithelium.

## 5. Conclusions

The secretome of *Bifidobacterium adolescentis* produces molecules and compounds capable of inhibiting rotavirus infectivity and possibly strengthening and defending the intestines from adverse events such as viral and bacterial infections that result from paracellular transport.

## Data Availability

Data will be provided upon request.

## References

[1] WHO Diarrhoeal Disease. https://www.who.int/news-room/fact-sheets/detail/diarrhoeal-disease.

[2] Caddy S., Papa G., Borodavka A., Desselberger U. (2021). Rotavirus research: 2014–2020. Virus Res..

[3] Desselberger U. (2014). Rotaviruses. Virus Res..

[4] Santos F.S., Sousa Junior E.C., Guerra S.F.S., Lobo P.S., Penha Junior E.T., Lima A.B.F., Vinente C.B.G., Chagas E.H.N., Justino M.C.A., Linhares A.C. (2019). G1P[8] Rotavirus in children with severe diarrhea in the post-vaccine introduction era in Brazil: Evidence of reassortments and structural modifications of the antigenic VP7 and VP4 regions. Infect. Genet. Evol..

[5] Rose T.L., Marques da Silva M., Goméz M.M., Resque H.R., Ichihara M.Y., de Mello Volotão E., Leite J.P.G. (2013). Evidence of vaccine-related reassortment of rotavirus, Brazil, 2008–2010. Emerg. Infect. Dis..

[6] Parashar U.D., Nelson E.A., Kang G. (2013). Diagnosis, management, and prevention of rotavirus gastroenteritis in children. BMJ.

[7] Steyer A., Mičetić-Turk D., Fijan S. (2022). The Efficacy of Probiotics as Antiviral Agents for the Treatment of Rotavirus Gastrointestinal Infections in Children: An Updated Overview of Literature. Microorganisms.

[8] Azagra-Boronat I., Massot-Cladera M., Knipping K., Garssen J., Ben Amor K., Knol J., Franch À., Castell M., Rodríguez-Lagunas M.J., Pérez-Cano F.J. (2020). Strain-Specific Probiotic Properties of Bifidobacteria and Lactobacilli for the Prevention of Diarrhea Caused by Rotavirus in a Preclinical Model. Nutrients.

[9] Ventola H., Lehtoranta L., Madetoja M., Simonen-Tikka M.L., Maunula L., Roivainen M., Korpela R., Holma R. (2012). Effects of the viability of Lactobacillus rhamnosus GG on rotavirus infection in neonatal rats. World J. Gastroenterol..

[10] Szajewska H., Setty M., Mrukowicz J., Guandalini S. (2006). Probiotics in gastrointestinal diseases in children: Hard and not-so-hard evidence of efficacy. J. Pediatr. Gastroenterol. Nutr..

[11] Ahmadi E., Alizadeh-Navaei R., Rezai M.S. (2015). Efficacy of probiotic use in acute rotavirus diarrhea in children: A systematic review and meta-analysis. Casp. J. Intern. Med..

[12] Pant N., Marcotte H., Brüssow H., Svensson L., Hammarström L. (2007). Effective prophylaxis against rotavirus diarrhea using a combination of Lactobacillus rhamnosus GG and antibodies. BMC Microbiol..

[13] Yang X., Twitchell E., Li G., Wen K., Weiss M., Kocher J., Lei S., Ramesh A., Ryan E.P., Yuan L. (2015). High protective efficacy of rice bran against human rotavirus diarrhea via enhancing probiotic growth, gut barrier function, and innate immunity. Sci. Rep..

[14] Erdoğan O., Tanyeri B., Torun E., Gönüllü E., Arslan H., Erenberk U., Oktem F. (2012). The comparition of the efficacy of two different probiotics in rotavirus gastroenteritis in children. J. Trop. Med..

[15] Cha M.K., Lee D.K., An H.M., Lee S.W., Shin S.H., Kwon J.H., Kim K.J., Ha N.J. (2012). Antiviral activity of Bifidobacterium adolescentis SPM1005-A on human papillomavirus type 16. BMC Med..

[16] Lee D.K., Kang J.Y., Shin H.S., Park I.H., Ha N.J. (2013). Antiviral activity of Bifidobacterium adolescentis SPM0212 against Hepatitis B virus. Arch. Pharm. Res..

[17] Botić T., Klingberg T.D., Weingartl H., Cencic A. (2007). A novel eukaryotic cell culture model to study antiviral activity of potential probiotic bacteria. Int. J. Food Microbiol..

[18] Kim M.J., Lee D.K., Park J.E., Park I.H., Seo J.G., Ha N.J. (2014). Antiviral activity of Bifidobacterium adolescentis SPM1605 against Coxsackievirus B3. Biotechnol. Biotechnol. Equip..

[19] Thorakkattu P., Khanashyam A.C., Shah K., Babu K.S., Mundanat A.S., Deliephan A., Deokar G.S., Santivarangkna C., Nirmal N.P. (2022). Postbiotics: Current Trends in Food and Pharmaceutical Industry. Foods.

[20] Fernandez-Duarte K.P., Olaya-Galán N.N., Salas-Cárdenas S.P., Lopez-Rozo J., Gutierrez-Fernandez M.F. (2018). Bifidobacterium adolescentis (DSM 20083) and Lactobacillus casei (Lafti L26-DSL): Probiotics Able to Block the In Vitro Adherence of Rotavirus in MA104 Cells. Probiotics Antimicrob. Proteins.

[21] Olaya Galán N.N., Ulloa Rubiano J.C., Velez Reyes F.A., Fernandez Duarte K.P., Salas Cárdenas S.P., Gutierrez Fernandez M.F. (2016). In vitro antiviral activity of Lactobacillus casei and Bifidobacterium adolescentis against rotavirus infection monitored by NSP4 protein production. J. Appl. Microbiol..

[22] OECD (2018). Guidance Document on Good In Vitro Method Practices (GIVIMP). https://www.oecd.org/env/guidance-document-on-good-in-vitro-method-practices-givimp-9789264304796-en.htm.

[23] Chelakkot C., Ghim J., Ryu S.H. (2018). Mechanisms regulating intestinal barrier integrity and its pathological implications. Exp. Mol. Med..

[24] Carrière V., Chambaz J., Rousset M. (2001). Intestinal responses to xenobiotics. Toxicol. Vitr..

[25] Simon-Assmann P., Turck N., Sidhoum-Jenny M., Gradwohl G., Kedinger M. (2007). In vitro models of intestinal epithelial cell differentiation. Cell Biol. Toxicol..

[26] Delaney B. (2017). In vitro studies with human intestinal epithelial cell line monolayers for protein hazard characterization. Food Chem. Toxicol..

[27] Peterson M.D., Mooseker M.S. (1992). Characterization of the enterocyte-like brush border cytoskeleton of the C2BBe clones of the human intestinal cell line, Caco-2. J. Cell Sci..

[28] Hidalgo I.J., Raub T.J., Borchardt R.T. (1989). Characterization of the human colon carcinoma cell line (Caco-2) as a model system for intestinal epithelial permeability. Gastroenterology.

[29] Anderson J.M., Van Itallie C.M. (1995). Tight junctions and the molecular basis for regulation of paracellular permeability. Am. J. Physiol..

[30] Anderson J.M., Van Itallie C.M. (2009). Physiology and function of the tight junction. Cold Spring Harb. Perspect. Biol..

[31] Trapecar M., Cencic A. (2012). Application of Gut Cell Models for Toxicological and Bioactivity Studies of Functional and Novel Foods. Foods.

[32] Lama S., Merlin-Zhang O., Yang C. (2020). In Vitro and In Vivo Models for Evaluating the Oral Toxicity of Nanomedicines. Nanomaterials.

[33] Roberts M.J., Bentley M.D., Harris J.M. (2012). Chemistry for peptide and protein PEGylation. Adv. Drug Deliv. Rev..

[34] Atha D.H., Ingham K.C. (1981). Mechanism of precipitation of proteins by polyethylene glycols. Analysis in terms of excluded volume. J. Biol. Chem..

[35] Zheng C., Ma G., Su Z. (2007). Native PAGE eliminates the problem of PEG-SDS interaction in SDS-PAGE and provides an alternative to HPLC in characterization of protein PEGylation. Electrophoresis.

[36] Téllez M.A., Téllez A.N., Vélez F., Ulloa J.C. (2015). In vitro antiviral activity against rotavirus and astrovirus infection exerted by substances obtained from Achyrocline bogotensis (Kunth) DC. (Compositae). BMC Complement. Altern. Med..

[37] Masuda K., Kajikawa A., Igimi S. (2011). Establishment and Evaluation of an in vitro M Cell Model using C2BBe1 Cells and Raji Cells. Biosci. Microflora.

[38] Reid G., Jass J., Sebulsky M.T., McCormick J.K. (2003). Potential uses of probiotics in clinical practice. Clin. Microbiol. Rev..

[39] Szajewska H., Mrukowicz J.Z. (2001). Probiotics in the treatment and prevention of acute infectious diarrhea in infants and children: A systematic review of published randomized, double-blind, placebo-controlled trials. J. Pediatr. Gastroenterol. Nutr..

[40] Allen S.J., Martinez E.G., Gregorio G.V., Dans L.F. (2010). Probiotics for treating acute infectious diarrhoea. Cochrane Database Syst. Rev..

[41] Kang J.Y., Lee D.K., Ha N.J., Shin H.S. (2015). Antiviral effects of Lactobacillus ruminis SPM0211 and Bifidobacterium longum SPM1205 and SPM1206 on rotavirus-infected Caco-2 cells and a neonatal mouse model. J. Microbiol..

[42] Lee D.K., Park J.E., Kim M.J., Seo J.G., Lee J.H., Ha N.J. (2015). Probiotic bacteria, B. longum and L. acidophilus inhibit infection by rotavirus in vitro and decrease the duration of diarrhea in pediatric patients. Clin. Res. Hepatol. Gastroenterol..

[43] Hsieh C.Y., Osaka T., Moriyama E., Date Y., Kikuchi J., Tsuneda S. (2015). Strengthening of the intestinal epithelial tight junction by Bifidobacterium bifidum. Physiol. Rep..

[44] Qiao H., Duffy L.C., Griffiths E., Dryja D., Leavens A., Rossman J., Rich G., Riepenhoff-Talty M., Locniskar M. (2002). Immune responses in rhesus rotavirus-challenged BALB/c mice treated with bifidobacteria and prebiotic supplements. Pediatr. Res..

[45] Vlasova A.N., Kandasamy S., Chattha K.S., Rajashekara G., Saif L.J. (2016). Comparison of probiotic lactobacilli and bifidobacteria effects, immune responses and rotavirus vaccines and infection in different host species. Vet. Immunol. Immunopathol..

[46] Gagnon M., Vimont A., Darveau A., Fliss I., Jean J. (2016). Study of the Ability of Bifidobacteria of Human Origin to Prevent and Treat Rotavirus Infection Using Colonic Cell and Mouse Models. PLoS ONE.

[47] An H.M., Lee D.K., Kim J.R., Lee S.W., Cha M.K., Lee K.O., Ha N.J. (2012). Antiviral activity of Bifidobacterium adolescentis SPM 0214 against herpes simplex virus type 1. Arch. Pharm. Res..

[48] Chenoll E., Rivero M., Codoñer F.M., Martinez-Blanch J.F., Ramón D., Genovés S., Moreno Muñoz J.A. (2015). Complete Genome Sequence of Bifidobacterium longum subsp. infantis Strain CECT 7210, a Probiotic Strain Active against Rotavirus Infections. Genome Announc..

[49] Araki K., Shinozaki T., Irie Y., Miyazawa Y. (1999). Trial of oral administration of Bifidobacterium breve for the prevention of rotavirus infections. Kansenshogaku Zasshi. J. Jpn. Assoc. Infect. Dis..

[50] Di Tommaso N., Gasbarrini A., Ponziani F.R. (2021). Intestinal Barrier in Human Health and Disease. Int. J. Environ. Res. Public Health.

[51] Ouwehand A.C., Salminen S., Isolauri E. (2002). Probiotics: An overview of beneficial effects. Antonie Van Leeuwenhoek.

[52] McFarland L.V., Evans C.T., Goldstein E.J.C. (2018). Strain-Specificity and Disease-Specificity of Probiotic Efficacy: A Systematic Review and Meta-Analysis. Front. Med..

[53] Yan F., Cao H., Cover T.L., Whitehead R., Washington M.K., Polk D.B. (2007). Soluble proteins produced by probiotic bacteria regulate intestinal epithelial cell survival and growth. Gastroenterology.

[54] Saberian M., Shahidi Delshad E., Habibi M. (2020). The Effect of Bifidobacterium Bifidum Supernatant and Cell Mass on the Proliferation Potential of Rat Bone Marrow-Derived Stromal Cells. Iran. J. Med. Sci..

[55] Ilinskaya O.N., Ulyanova V.V., Yarullina D.R., Gataullin I.G. (2017). Secretome of Intestinal Bacilli: A Natural Guard against Pathologies. Front Microbiol..

[56] Tao Y., Drabik K.A., Waypa T.S., Musch M.W., Alverdy J.C., Schneewind O., Chang E.B., Petrof E.O. (2006). Soluble factors from Lactobacillus GG activate MAPKs and induce cytoprotective heat shock proteins in intestinal epithelial cells. Am. J. Physiol. Cell Physiol..

[57] Srinivasan B., Kolli A.R., Esch M.B., Abaci H.E., Shuler M.L., Hickman J.J. (2015). TEER measurement techniques for in vitro barrier model systems. J. Lab. Autom..

[58] Méry B., Guy J.B., Vallard A., Espenel S., Ardail D., Rodriguez-Lafrasse C., Rancoule C., Magné N. (2017). In Vitro Cell Death Determination for Drug Discovery: A Landscape Review of Real Issues. J. Cell Death.

[59] Khailova L., Mount Patrick S.K., Arganbright K.M., Halpern M.D., Kinouchi T., Dvorak B. (2010). Bifidobacterium bifidum reduces apoptosis in the intestinal epithelium in necrotizing enterocolitis. Am. J. Physiol. Gastrointest. Liver Physiol..

[60] Macko P., Palosaari T., Whelan M. (2021). Extrapolating from acute to chronic toxicity in vitro. Toxicol. Vitr..

